# A comprehensive review of mechanisms underlying resistance to immune checkpoint inhibitors

**DOI:** 10.3389/fimmu.2026.1735741

**Published:** 2026-03-03

**Authors:** Alexandre Bertucci, Khaoula Taleb, Emilien Billon, Téo Fernez, Philippe Rochigneux

**Affiliations:** 1 Department of Medical Oncology, Paoli-Calmettes Institute, Marseille, France; 2 Team Immunity and Cancer, Centre de Recherche en Cancérologie de Marseille (CRCM), INSERM U1068, CNRS UMR 7258, Aix-Marseille University UM105 and Paoli-Calmettes Institute, Marseille, France

**Keywords:** acquired resistance, immunosuppression, immunotherapy, neoantigen machinery defects, primary resistance, tumour micro environment

## Abstract

Immune checkpoint inhibitors (ICIs) have revolutionized the management of multiple malignancies, offering durable clinical benefit in a subset of patients. However, the emergence of distinct response patterns has revealed two major challenges: primary resistance, observed in patients who fail to respond from the outset, and acquired resistance, which develops after an initial period of disease control. These two resistance phenotypes likely arise from divergent biological mechanisms, involving both tumour-intrinsic and tumour-extrinsic factors. A comprehensive understanding of these processes is essential to optimize therapeutic strategies, particularly through rational combinations of ICIs with novel immunomodulators, targeted therapies, or conventional treatments. In this review, we provide an integrative overview of the key molecular and cellular mechanisms underlying both primary and acquired resistance to ICIs, encompassing alterations in antigen presentation, interferon signalling, oncogenic and metabolic pathways, as well as immune exclusion within the tumour microenvironment. We also highlight emerging predictive biomarkers of response and resistance—ranging from genomic and transcriptomic signatures to soluble immune checkpoints and non-immune circulating markers—aimed at refining patient selection and guiding personalized immunotherapy. Ultimately, deciphering these mechanisms will be pivotal for designing the next generation of immune-based combinations to overcome therapeutic resistance and expand the population of patients who can benefit from immune checkpoint blockade.

## 1 Introduction

Immune checkpoint inhibitors (ICIs) have transformed cancer care, enabling prolonged complete response in some metastatic settings, and showing efficacy in localized disease. Yet, some patients did not benefit of ICIs or ultimately experience progression after treatment initiation or discontinuation. Tumour cells evade immune surveillance by establishing immunosuppressive microenvironments and escaping recognition, leading to either primary or acquired resistance. Primary resistance reflects a failure to respond upfront, while acquired resistance involves immune recognition followed by tumour adaptation—sometimes presenting clinically as primary or acquired resistance (1).

Acquired resistance (AR), in contrast, emerges after an initial response and is defined variably across studies and tumour types (2–4). Definitions remain debated, particularly regarding stable disease, treatment duration, and ICIs discontinuation. Efforts to standardize these definitions have been made by Sharma et al., IASLC, and Society for Immunotherapy of Cancer (SITC) (5–7), yet further refinement is needed through clinical datasets to support consistent classification and guide therapeutic decisions.

AR is biologically distinct from primary resistance in different aspect. First, the immunophenotype, AR typically arises in inflamed tumours via co-evolution with the tumour microenvironment (TME), whereas primary resistance often reflects a non-inflamed (cold) tumour. Therapeutic approaches to AR focus on mechanism-specific interventions, while primary resistance strategies often aim to inflame the tumour (8, 9). Second, the underlying mechanisms: AR can be attributed to the co-evolution of the tumour and the TME under the influence of the immune elimination activated by ICIs therapy. In contrast, primary resistance may be attributed to a combination of factors involving the host, tumour, TME, and microbiome, reflecting the baseline resistance to ICIs (10, 11). And third, the coping strategies: The strategies employed to address AR may be specific to the mechanisms of resistance and are aimed at prolonging the survival of patients. In contrast, the coping strategies for primary resistance are typically more comprehensive and often revolve around “heating” the cold tumour, a term used to describe the process of turning non-inflamed or poorly infiltrated tumours into immunologically active ones (9).

Clinically, AR remains under-characterized across tumour types. Reported rates range from 11% to 71%, with a median of 39%, and are higher in tumours with initially low response rates. AR to nivolumab, an anti-programmed death 1 (PD-1) in melanoma was reported to be 39% at a 5-year follow-up (12), while AR to nivolumab in non-small-cell lung cancer (NSCLC) reached 65% at a 4-year follow-up (13). Duration of response also varies, melanoma shows the longest median duration of response (>4 years), whereas other cancers typically relapse within two years. AR occurs more frequently after partial response than complete responses, and oligo-progression is a common pattern. However, inconsistencies in AR definitions across studies complicate interpretation (13, 14).

Two main biological modes of AR have been proposed: Darwinian selection and homeostatic resistance. In the Darwinian model, pre-existing resistant clones survive immune pressure, correlating inversely with tumour size and displaying impaired cytotoxic T cell responses despite intact MHC-I and interferon-gamma (IFN-γ) pathways (15, 16). In the homeostatic model, resistance emerges *de novo* in initially sensitive cells through immune adaptation. Tumour cells exhibit a remarkable ability to reprogram their biology in response to drug-induced immune pressure. Under ICIs treatment this acquired response is characterized by the upregulation of PD-L1 expression triggered by IFN-γ released from activated immune cells. This molecular shift enables tumour cells to escape immune surveillance and develop resistance to ICIs (17).

Despite their conceptual clarity, these mechanisms remain difficult to disentangle. Whether resistance arises from pre-existing traits or single-cell adaptations is still debated. Single-cell and spatial multi-omics now enable high-resolution insights into these dynamics (18, 19). Darwinian evolution appears predominant, though the balance between mechanisms remains uncertain. Importantly, these models often overlook the role of immune cells. Categorizing resistance as tumour-intrinsic or -extrinsic enables a more comprehensive framework for analysis (20, 21).

In this Review, we summarize current understanding of immune evasion and resistance to ICIs, integrating tumour-intrinsic and -extrinsic mechanisms.

## 2 Intrinsic mechanism of resistance to immune checkpoint inhibitors

**Figure 1:**
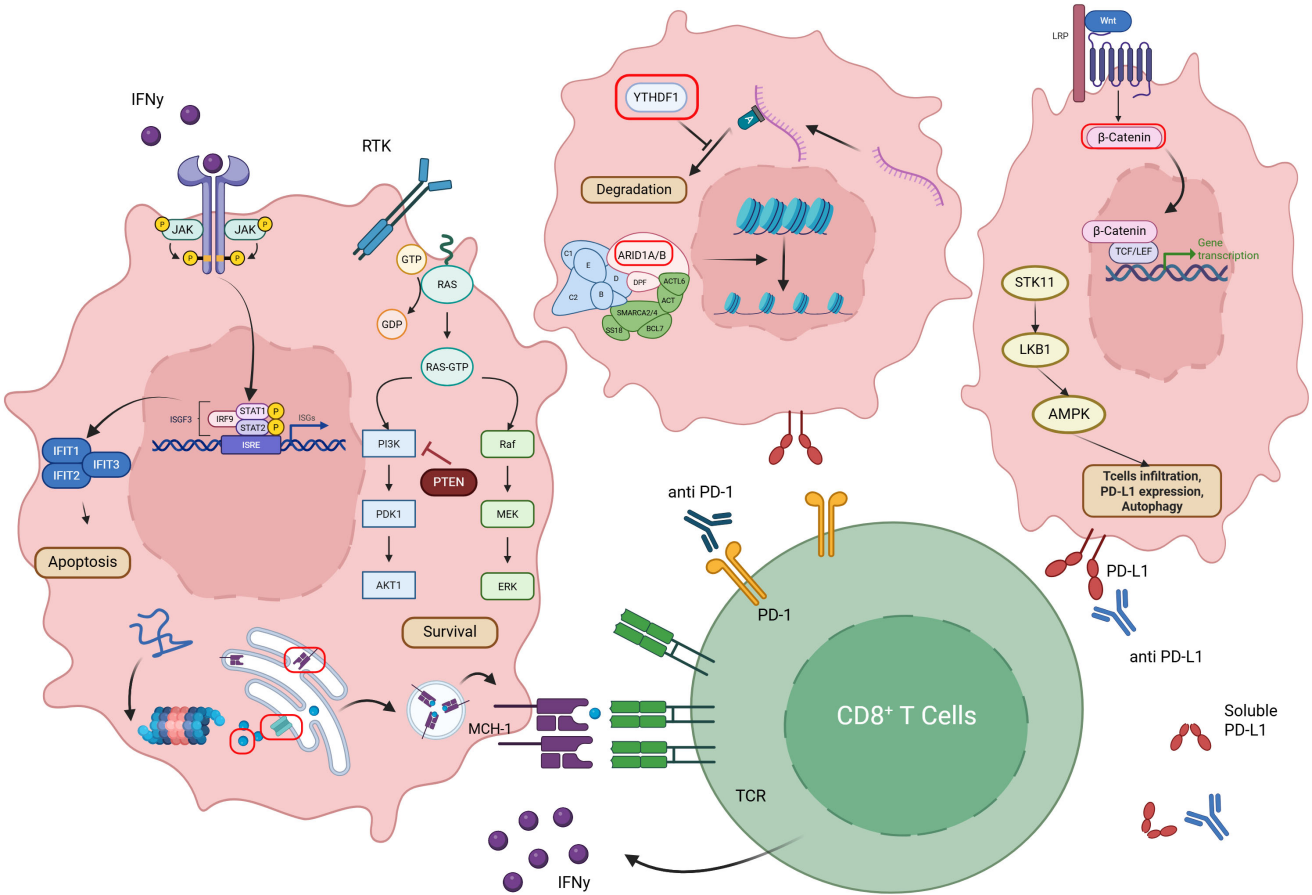
Intrinsic tumour-cell mechanisms that impair antigenicity and promote tumour immune evasion. Schematic of cell-intrinsic pathways within a representative cancer cell that reduce antigen presentation, increase survival and antagonise cytotoxic T-cell responses. Oncogenic signalling (e.g., RAS) activates parallel RAF–MEK–ERK and PI3K–PDK1–AKT cascades to promote proliferation and survival, while the lipid-phosphatase PTEN antagonises PI3K signalling. Wnt–β-catenin and JAK–STAT programmes drive transcriptional changes that limit anti-tumour immunity (including regulation of interferon-stimulated genes such as IFIT1/2/3 and the ISGF3 complex acting at ISREs). Loss or alteration of chromatin-remodelling components (SWI/SNF complex subunits such as ARID1A/B, SMARCA2/4 and associated factors) and post-transcriptional regulators (e.g., the m6A reader YTHDF1) modify expression, stability and translation of transcripts required for antigen processing and presentation. Defects in proteasomal degradation, peptide transport/ loading and ER–Golgi trafficking reduce major histocompatibility complex class I (MHC-I) surface display. Cellular stress and metabolic regulators (STK11/LKB1 → AMPK) influence autophagy, PD-L1 expression and the tumour metabolic microenvironment, thereby altering T-cell infiltration and function. Upregulation of PD-L1 on tumour cells engages PD-1 on T cells to inhibit effector activity. Arrows indicate the principal direction of influence between modules; T-bars denote suppressive interactions on antigenicity or immune activation.

High-quality neoantigens are critical for the effectiveness of ICIs, and their loss represents a key mechanism of immune resistance. Under immune pressure, cancer cells may evade detection via HLA loss of heterozygosity (LOH) or direct neoantigen depletion (22, 23). In tumours with intact HLA and high immune infiltration, this depletion often reflects active immunoediting, whereby immunogenic clones are selectively eliminated by the immune system. While this process indicates a functional immune response, it paradoxically contributes to acquired resistance by reducing the pool of targetable neoantigens and impairing long-term efficacy of immunotherapy. In contrast, tumours harbouring HLA LOH tend to accumulate subclonal neoantigens that escape immune recognition due to impaired antigen presentation. These distinct trajectories reflect context-dependent mechanisms of immune evasion (24). **Figure 1** and **Table 1** summarized the major intrinsic mechanism of resistance.

**Table 1:** Summary of the main intrinsic resistance mechanisms to immune checkpoint inhibitors.

Mechanism	Main pathway	Primary resistance	Acquired resistance	Predictive value	Target	Number of clinical trial	Phase	Type of cancer
Clonal neoantigens	Ag presentation	**++**	**+**	X	RT	1273	4	Pan cancer
Silencing neoantigens	Ag presentation	**-**	**+**	X				
Loss of neoantigens	Ag presentation	**-**	**+**	0				
MHC-1 or β2M loss	Ag presentation	**+**	**++**	X				
MHC-1 or β2M decrease	Ag presentation	**+**	**++**	X	HDAC or DNMT STING / TLR	HDAC: 11 Sting: 1 TLR: 1	2 2 1	HDAC: Pan cancer Sting: NCSLC & RCC TLR: Melanoma
IFNγ pathway	T cell response	**-/+**	**+**	0	IFNγ	1	2	Sarcoma
JAK1/2 mutation	T cell response	**-**	**+**	0	JAK1/2	3	2	Breast cancer Lymphoma
AKT1/CDH1 mutation	Cell cycle	**+**	**-**	X	AKT1	AKT: 2	2	Breast cancer
PTEN loss	Cell cycle	**-/+**	**+**	0	PI3K	4	2	NSCLC Lymphoma CLL
PIK3CA mutation	Cell cycle	**+**	**-/+**	0	PI3K	4	2	NSCLC Lymphoma CLL
STK11 mutation	Cell cycle	**+**	**-**	0				
WNT/ß catenin	Cell Cycle	**+**	**-**	0				
CCND1 amplification	Oncogene	**+**	**-**	0	CDK4/6	9	2	Pan cancer
KRAS mutation	Oncogene	G12D +	**-**	X	KRAS G12D	0		
SWI/SNF complex	Epigenetic	**+**	**-**	0	EZH2 or ATR	EZH2: 2 ATR: 6	2 2	EZH2: NSCLC/gastric cancer ATR: Pan cancer
YTHDF1	Epigenetic	**-/+**	**-/+**	0				
Chemokines pathway	Cell comm.	**+**	**+**	0	CCR5 (ex. Maraviroc)	2	2	PDAC CRC

RT, Radiotherapy; MHC-I, Major Histocompatibility Complex class I; β2M, Beta-2 Microglobulin; HDAC, Histone Deacetylase; DNMT, DNA Methyltransferase; STING, Stimulator of Interferon Genes; TLR, Toll-Like Receptor; IFNγ, Interferon gamma; JAK1/2, Janus Kinase 1 and 2; AKT1, AKT Serine/Threonine Kinase 1; PTEN, Phosphatase and Tensin Homolog; PI3K, Phosphoinositide 3-Kinase; PIK3CA, Phosphatidylinositol-4, 5-Bisphosphate 3-Kinase Catalytic Subunit Alpha; STK11 (LKB1), Serine/Threonine Kinase 11; WNT, Wingless-related integration site; β-catenin, Beta-catenin (CTNNB1); CCND1, Cyclin D1; CDK4/6, Cyclin-Dependent Kinase 4 and 6; KRAS, Kirsten Rat Sarcoma Viral Oncogene Homolog; SWI/SNF, SWItch/Sucrose Non-Fermentable chromatin-remodeling complex; EZH2, Enhancer of Zeste Homolog 2; ATR, Ataxia Telangiectasia and Rad3-related kinase; YTHDF1, YTH N6-Methyladenosine RNA Binding Protein 1; CCR5, C-C Chemokine Receptor type 5; NSCLC, Non-Small Cell Lung Cancer; RCC, Renal Cell Carcinoma; CRC, Colorectal Cancer; PDAC, Pancreatic Ductal Adenocarcinoma; CLL, Chronic Lymphocytic Leukemia.

### 2.1 Antigen presentation

#### 2.1.1 Clonal neoantigens and immune response

Clonal neoantigens—shared across a majority of tumour cells—are pivotal to durable immune responses post-ICIs. A dichotomy exists between tumour-specific antigens (TSAs) and tumour-associated antigens (TAAs). TSAs arise from somatic mutations that generate neoantigens—mutant proteins absent from healthy tissues (e.g., BCR-ABL or mutated BRAF). In contrast, TAAs result from epigenetic dysregulation, hyperproliferation, transcriptional alterations, or gene amplification, leading to the aberrant overexpression of otherwise normal proteins (e.g., PSA or β-catenin). Their significance stems from several factors:

- Minimum Cell Fraction Threshold: Effective T cell–mediated rejection may require neoantigens to be present in a substantial fraction of tumour cells (23).
- Broader Immune Control: Clonal neoantigens, due to widespread expression across the tumour mass, enhance the likelihood of complete immune recognition.
- Allelic Fraction Amplification: In tumours such as NSCLC, genome doubling increases allelic frequency of early-arising neoantigens, potentially enhancing T cell visibility.
- Predictive Correlation: The clonal neoantigen burden correlates positively with response to ICIs (22).

Neoantigens with low similarity to self-antigens are more likely to elicit immune responses. Recent studies suggest that frameshift insertions, especially those creating novel open reading frames (neoORFs), are a potent source of immunogenic peptides (25). However, the role of sequence homology in enhancing recognition remains under investigation. Neoantigen potency may depend on the underlying mutational process, with mismatch repair deficiency potentially yielding more immunostimulatory mutations than processes like cytosine deamination.

#### 2.1.2 Transcriptional silencing of neoantigens

Neoantigens must be transcribed and translated to activate immunity. Purifying selection on neoantigens within genes typically expressed in lung cancer highlights the importance of expression context (26). In early-stage NSCLC (TRACERx cohort), approximately 20% of non-expressed neoantigens were subject to promoter methylation despite high predicted HLA affinity. Other silencing mechanisms—including exon skipping and chromatin remodelling—can likewise suppress immunogenic transcripts and must be accounted for in neoantigen discovery.

#### 2.1.3 Loss of neoantigen

##### 2.1.3.1 Tolerance of neoantigen loss and evolutionary selection

Most neoantigens are passenger mutations; their loss via chromosomal instability often incurs minimal fitness cost. However, deletion of essential neoantigens—mutations in viability-critical genes—can impair tumour survival. These essential neoantigens, particularly when located in haploid LOH regions, may represent potent immune targets due to the constraints against their loss. Neoantigen loss has been observed during ICIs. In NSCLC, resistant tumours exhibited loss of 7–18 putative neoantigens present at baseline (2). Corresponding autologous T cells had previously responded to peptides from these neoantigens, suggesting immunological selection pressure. Resistance arose from subclone elimination or chromosomal deletions of truncal mutations, with associated shifts in T cell receptor (TCR) clonality—supporting immune editing of mutation-associated neoantigens (MANAs) as a resistance mechanism. In a head and neck squamous cell carcinoma case, two candidate neoantigens were lost post-ICIs; one, HSPA12B P>R, was deleted via chromosome 20 LOH (2). These data suggest that immunoediting-driven depletion of clonal neoantigens limits durable responses to ICIs (2).

##### 2.1.3.2 Neoantigen loss across tumour types and therapies

In NSCLC, patients with AR to ICIs exhibited loss of predicted high-affinity neoantigens, without notable changes in PD-1, PD-L1, CTLA-4 expression, or immune-related genomic alterations. These neoantigens stimulated patient-derived T cells *in vitro*, reinforcing their immunogenicity. Similar findings were reported in a case of uterine leiomyosarcoma, where resistance to PD-1 blockade involved the reduction of immunogenic neoantigen expression, alongside bi-allelic PTEN loss (27).

Neoantigen depletion is also implicated in resistance to T cell transfer therapies (28, 29). This can arise from Darwinian selection eliminating neoantigen-bearing clones, or from reversible transcriptional adaptations. While the former is likely inevitable under immune pressure, the latter may be therapeutically targetable, like epigenetic drug as hypomethylating agent.

##### 2.1.3.3 Genomic context matters

The chromosomal location of a neoantigen can influence its persistence. Neoantigens arising in essential genes—especially within genomic regions refractory to deletion—may offer greater stability and immunotherapeutic potential. Understanding the interplay between mutation type, transcriptional regulation, and genomic context is essential for identifying robust immunogenic targets.

#### 2.1.4 Disruption of antigen presentation

Effective antigen presentation via MHC-I is essential for cytotoxic T lymphocyte (CTLs)–mediated tumour clearance. Disruptions in this process, including HLA-I loss, β2-microglobulin (ß2M) mutations, or TAP deficiency, enable immune evasion (30).

##### 2.1.4.1 MHC-I loss and its consequences

Loss of heterozygosity (LOH) at the HLA locus often reflects the targeted deletion of the allele responsible for presenting immunogenic neoantigens. Under immune pressure, tumours frequently acquire mutations or transcriptional silencing in antigen-presenting genes, particularly in the TCR-binding domains of HLA molecules. Reduced or absent HLA-I expression is a well-documented immune escape strategy, associated with poor prognosis and diminished response to PD-1/PD-L1 inhibitors across multiple cancers (31, 32). While PD-L1 expression and TILs presence correlate positively with ICIs efficacy (33), concurrent loss of HLA-I may neutralize these advantages. Tumours exhibiting a HLA-I^–^/PD-L1^+^ phenotype demonstrate resistance to CTLs and represent a form of acquired immune evasion (34). In NSCLC, dual assessment of HLA-I and PD-L1 expression has prognostic and predictive implications. Tumours with HLA-I positivity showed significantly more CD45RO^+^ memory T cells compared to HLA-I^–^ counterparts (p < 0.007), while CD45, CD3, and FoxP3^+^ Tregs were comparable (35). HLA-I^–^/PD-L1^+^ tumours exhibited greater primary extension, lymphatic spread, and lower CD8^+^ T cell infiltration than HLA-I^+^/PD-L1^+^ tumours (p < 0.023). Interestingly, HLA-I^+^/PD-L1^–^ tumours were most frequently found in early-stage disease (64%, stage I), suggesting an immune-responsive phenotype. Topographical analysis revealed that HLA-I^+^/PD-L1^+^ tumours often displayed intra-tumoural CD8^+^ infiltration, while HLA-I^–^/PD-L1^+^ tumours showed peripheral/stromal localization (p < 0.003) (24). Loss of HLA-I expression, frequently associated with LOH on chromosome 6, reflects tumour adaptation to immune pressure. Flow cytometry data from NSCLC cell lines showed variability in surface HLA-I expression, with four of six lines exhibiting partial or total HLA haplotype loss. One line (SK-MES) also demonstrated LOH affecting both class I and II loci and IFN-γ unresponsiveness (36). Despite HLA polymorphism complexity, some HLA-I mutations appear recurrent and selected under immune pressure. HLA LOH has been inconsistently associated with ICIs resistance, though HLA-corrected tumour mutational burden (TMB) shows improved predictive value over raw TMB. Based on a large dataset, HLA-I loss is observed in 17% in a pan-cancer cohort (n=83,644). In no squamous NSCLC treated with ICI, HLA-I loss was associated to worse overall survival (37).

In one case of TCR-T therapy targeting KRAS, tumour relapse was linked to deletion of the HLA-C*08:02 haplotype involved in antigen presentation (38). Similar cases of AR to ICIs and T cell therapy have shown selective transcriptional silencing of HLA alleles following treatment (39, 40). HLA-I loss may also reflect epigenetic regulation, influenced by TGF-β or PRC2 activity, and is associated with MITF^low^/AXL^high^ dedifferentiation and fibroblast signatures. Additional factors such as HPV16 E5, IL-8, and autophagy may further disrupt antigen processing (41–43).

By eliminating the HLA allele that binds and displays specific tumour-derived peptides, cancer cells can evade CD8^+^ T cell recognition while retaining the non-presenting allele, thereby preserving partial MHC-I functionality and avoiding natural killer (NK) cell activation.

##### 2.1.4.2 ß-microglobulin loss and its consequences

The MHC-I complex requires ß-Microglobulin *(*ß2M) for proper folding and surface expression. Loss of ß2M abolishes antigen presentation to CD8^+^ T cells. A 4-bp frameshift mutation in ß2M was identified in paired pre- and post-treatment biopsies from a patient with AR; IHC confirmed lack of membrane-bound MHC-I despite intracellular accumulation (44, 45). MHC-II expression was also absent. In a broader cohort, baseline ß2M LOH was more frequent in ICIs-resistant tumours (28.9%) than in responders (11.1%) (p = 0.03). Notably, ß2M LOH increases the likelihood—but does not guarantee—ß2M protein loss. In some patients, ß2M LOH coexists with ICIs sensitivity, suggesting additional mechanisms (e.g., epigenetic silencing) influence outcomes. Contradictory data exist: some studies found ß2M loss in ICIs responders, particularly in microsatellite instability–high (MSI-H) colorectal cancers, where ß2M status did not correlate with TILs density. ICIs response may be preserved in MSI-H tumours with ß2M loss, possibly due to alternative antigen presentation pathways (antigen presentation through MHC class II molecules activates CD4^+^ T cells, which subsequently recruit and enhance the cytotoxic activity of CD8^+^ T cells) Cross-presentation allows dendritic cells to capture tumour antigens and present them via MHC class I to prime CD8^+^ T-cell responses). Nevertheless, other reports highlight ß2M loss as a driver of resistance, even in dMMR/MSI-H contexts. In a longitudinal melanoma case (Ma-Mel-48), progression from T cell–responsive to T cell–resistant tumours involved progressive loss of HLA-I expression, culminating in a bi-allelic ß2M mutation and total loss of MHC-I presentation. The deletion of chromosome 15q21.1, encompassing ß2M, is not uncommon in melanoma and contributes to immune escape (46, 47). Five metastatic melanoma–derived cell lines from ICIs-treated patients showed impaired CD8^+^ T cell recognition due to ß2M loss. Reintroducing ß2M restored antigen presentation and T cell recognition in HLA-A2.1–matched cells. Given MHC-I instability without ß2M, this loss is a particularly potent immune escape route. While TAP or proteasome component loss reduces antigen processing, only ß2M loss fully abolishes MHC-I function (48, 49).

Loss of ß2M may sensitize tumours to NK cell–mediated lysis, yet clinical progression of ß2M-deficient tumours suggests additional escape strategies against innate immunity (50). Bi-allelic inactivation—whether by deletion or mutation—represents a critical checkpoint in acquired immune resistance.

### 2.2 T cell response

#### 2.2.1 IFN gamma pathway

Loss of IFN-γ pathways deteriorates T cell responses and allows tumour growth (51, 52). The function of IFN-γ signalling in tumour cells in the context of anti-CTLA-4 is still unclear. Non-responder cancers have chromosomal deletions in downstream IFN-γ pathway genes such IFIT1, IFIT2, and IFIT3 gene families. These genes promote tumour apoptosis (53). Anti-CTLA-4 improved T cell IFN-γ production, *in vivo*, and that IFN-γ signalling in T cells is crucial for the anti-tumour immune response mediated by anti-CTLA-4 (54, 55). By binding to the IFN-γ receptor and then activating the JAK-STAT signalling cascade, *in vitro* treatment with IFN-γ can directly suppress tumour cell proliferation and induce tumour cell apoptosis in addition to activating immune cells (51).

Melanoma samples from patients who progressed with ipilimumab have a significantly greater proportion of genetic abnormalities in the genes involved in the IFN-γ pathway. With the suppression of IFNGR1 expression in the B16/BL6 melanoma tumour cell lines, it was found that these cells were insensitive to IFN-γ *in vitro*. Additionally, mice given anti-CTLA-4 showed decreased rejection of B16/BL6 tumours lacking expression of the IFNGR1 gene in comparison to mice bearing B16/BL6 tumours with intact expression of the IFNGR1 gene (56).

#### 2.2.2 Mutation of JAK1/2

In a study involving four melanoma patients who exhibited AR to pembrolizumab, the resistant tumours of two patients were discovered to harbour truncating mutations in JAK1 or JAK2, combined with LOH. Importantly, no JAK1/2 mutations were detected in the baseline tumours in these patients. Functionally, this loss of JAK1/2 function resulted in defects in IFN-γ induced growth arrest, MHC-I expression, PD-L1 expression and antigen presentation (3). Furthermore, these mutations are also associated with primary resistance to anti–PD-1 in melanoma and mismatch repair–deficient cancers (57). Additionally, genomic analysis unveiled that the loss of the tumour suppressor *CDKN2A* can enhance the susceptibility of *JAK2* loss, thereby increasing the likelihood of AR (58). While JAK1 loss-of-function is strongly associated with resistance to anti–PD-L1 therapy due to impaired IFN-γ signalling, *JAK2* deficiency alone may not confer the same level of immune escape. Indeed, recent findings suggest that metastases harbouring *JAK2* loss-of-function mutations can still respond to immune checkpoint inhibitors, highlighting a functional distinction between *JAK1* and *JAK2* in regulating tumour immunogenicity (59).

### 2.3 Cell cycle

#### 2.3.1 AKT1 and CDH1 mutations

Mutations in *AKT1* and *CDH1* have been found to be an independent predictor of poor progression-free survival (PFS) and initial ICIs resistance in dMMR/MSI-H gastro-intestinal (GI) cancer. With high performance in predicting primary resistance, Wang et al. (60) combined these two genes to create an immuno-oncology therapy predictor (IOpred). Patients with IOpred-Mut GI cancer had significantly shorter PFS (HR = 8.36, p<0.001) and OS (HR = 5.17, p<0.001) and worse prognosis (PFS, HR = 4.68, p=0.004; OS, HR = 15.98, p<0.001) after ICI treatment than IOpred-WT patients. In the validation cohort, IOpred’s positive predictive value for detecting primary resistance to ICIs was 80%, while its sensitivity is moderate with 20% of IOpred-WT patients have primary resistance to ICIs. When compared to ICIs-sensitive subgroup, the *AKT1* and *CDH1* mutations were considerably more prevalent in the ICIs-resistant cohort. When compared to *AKT1* wild type tumours, *AKT1* mutated tumours had a considerably lower M1/M2 ratio of tumour-associated macrophages.

#### 2.3.2 PTEN loss

*PTEN* loss contributes to immune resistance by shaping an immunosuppressive TME, notably through PTPN11 upregulation and Jak2/Stat3 activation in prostate cancer (61), though its role in resistance remains unclear in melanoma and NSCLC (62). Peng et al. showed that *PTEN* deletion reduces T cell infiltration and tumour cell killing via increased immunosuppressive cytokines and autophagy suppression (63). In mouse models, combining PI3K inhibitors with ICIs enhanced anti-tumour responses. Clinically, biallelic PTEN loss has been reported in resistant tumours from patients with melanoma or uterine leiomyosarcoma treated with anti-PD-1 or anti-CTLA-4 inhibitors (64). In a melanoma cohort with AR, *PTEN* loss and β-catenin activation emerged as non-overlapping resistance mechanisms (65), highlighting the heterogeneity of immune escape. These findings support evaluating PI3K–AKT–mTOR pathway inhibitors in combination with ICIs.

#### 2.3.3 PIK3CA mutations

The PI3K inhibitor LY294002 has been demonstrated to prevent the growth of *PIK3CA*-mutant colon cancer by downregulating PIK3CA signalling (66). A combination of PI3K inhibitor and ICIs may increase the efficacy of ICIs in patients with defective antigen presentation, which may be a trait conferred by *PIK3CA* mutations. In the interim, *PIK3CA* mutations may increase TMB (67).

#### 2.3.4 STK11 mutations

In both human cancers and genetically modified murine models, inactivation of STK11 by mutational or non-mutational processes correlated with an inactive or “cold” tumour immune microenvironment, with lower density of infiltrating cytotoxic CD8^+^ T cells (68–70). STK11 (serine/threonine kinase 11), also known as LKB1, is a tumour suppressor that regulates cellular metabolism, polarity, and stress responses, and plays a key role in maintaining immune homeostasis within the tumour microenvironment. Skoulidis et al., identified STK11/LKB1 inactivation as a major tumour-intrinsic driver of primary resistance to PD-1 blockade in KRAS-mutant NSCLC (71). STK11/LKB1-deficient tumours display low PD-L1 expression and reduced CD8^+^ T cell infiltration, despite intermediate or high TMB. Resistance extends to PD-L1–positive tumours, indicating partial PD-L1 independence. In PD-L1–negative *KRAS*-mutant NSCLC, some tumours still showed partial responses or stable disease, suggesting heterogeneous immune responses. STK11 loss-of-function mutations confer resistance to durvalumab ± tremelimumab in NSCLC by suppressing IFN-γ signalling and CD8^+^ T cell infiltration. *STK11*-mutant tumours show upregulation of STAT3 and reduced MHC-I expression. STAT3 knockdown restores antigen presentation and enhances T cell–mediated cytotoxicity. *In vivo*, STAT3 inhibition re-sensitizes *STK11*-mutant tumours to PD-L1 blockade (72).

Together, these findings support the role of STK11/LKB1 loss as a central mediator of immune escape and resistance to ICIs in *KRAS*-mutant NSCLC, involving multiple non-overlapping and converging pathways that shape a cold TME (73).

#### 2.3.5 WNT/ß catenin

The Wnt/β-catenin signalling pathway is implicated in immune evasion. Activation of the Wnt/β-catenin pathway within the tumour triggers a cascade of events that significantly hinders the production of CCL4, a chemokine released by CD103^+^ dendritic cells. This, in turn, exerts inhibitory effects on the activation and infiltration of CD8^+^ T cells (74, 75).

### 2.4 Oncogenes

#### 2.4.1 Focal amplification of *CCND1* associates with ICIs resistance

*CCND1* encodes Cyclin D1, which binds CDK4/6 to phosphorylate the retinoblastoma protein (Rb), thereby releasing E2F transcription factors and promoting G1/S cell cycle progression. This amplification drives uncontrolled proliferation and may contribute to immune evasion. Significantly reduced rates of ICIs response in tumours with *CCND1* amplification compared to wild-type were highly associated. Prior functional data shows a role for *CCND1* in determining ICIs response (76). The majority of *CCND1* amplified tumours were found in urothelial carcinomas, thus it was demonstrated in the mRNA levels that non-responders to treatment for urothelial cancer express *CCND1* at considerably greater levels than do responders. *CCND1* amplification was associated with reduced overall survival (OS), with a large effect size supporting its prognostic impact (77). However, in the MSK-IMPACT urothelial carcinoma cohort, no OS difference was observed between amplified and non-amplified tumours, indicating that the prognostic value of *CCND1* may be context-dependent.

#### 2.4.2 KRAS mutation

ICIs are less effective in NSCLC with *KRAS*-G12D mutations in advanced NSCLC. Mechanistically, these mutations suppress PD-L1 expression via the P70S6K/PI3K/AKT signalling axis and downregulate HMGA2, leading to decreased production of CXCL10 and CXCL11—key chemokines for T cell recruitment. Notably, paclitaxel has been shown to restore HMGA2 expression and chemokine secretion, potentially reversing the immunosuppressive phenotype. *KRAS* mutations correlate with lower CD8^+^ TILs, reduced TMB, and diminished PD-L1, contributing to an immune-cold tumour and resistance to ICIs (78).

Recent studies indicate KRAS mutations are not reliable predictors of ICI response in advanced NSCLC (79, 80). However, different KRAS variants have distinct effects: G12C associates with higher PD-L1, while G12V enhances PD-L1 via EMT/TGF-β signalling (81). In pancreatic cancer, KRAS-G12D drives Treg differentiation through MEK/ERK signalling pathway (82). In colorectal cancer, the same mutation promotes myeloid suppressor cell recruitment via the IRF2–CXCL3–CXCR2 axis (83), establishing an immunosuppressive TME and fostering ICIs resistance. Together, these findings suggest KRAS-G12D mutations shape immune evasion through multiple, context-specific mechanisms.

### 2.5 Epigenetic and post-transcriptional regulation of immune responsiveness

#### 2.5.1 Chromatin remodelling (SWI/SNF complex)

AT-Rich Interactive Domain-containing protein 1A (*ARID1A*) or 1B (*ARID1B*), stands out as the most common target of mutations within the SWI/SNF complex. The SWI/SNF complex regulates chromatin accessibility and gene expression by repositioning nucleosomes and is essential for maintaining genomic stability and cell identity. Mutations in *ARID1A* can destabilize the SWI/SNF complex, compromising its function and affecting tumour cell viability. The concurrent mutation of both *ARID1A* and *ARID1B* proves to be synthetically lethal in colon cancer. *ARID1A* mutations were significantly more frequently associated with immunological infiltration in colon cancer. Moreover, MSH2 interacts with ARID1A. Increases in TMB and cytotoxic T cell infiltration are due to low expression of ARID1A, which impairs DNA mismatch repair (84). Although *ARID1A* mutations are not currently established as predictive biomarkers of response to ICIs, their association with increased immune infiltration in colorectal cancer suggests a potential link to tumour immunogenicity. The observed increase in cytotoxic T cell infiltration and TMB, likely driven by impaired mismatch repair, may contribute to a more inflamed tumour microenvironment. These features could influence sensitivity to ICIs, but further studies are needed to clarify whether ARID1A loss directly enhances immunotherapy efficacy.

#### 2.5.2 YTHDF1

Excessive tumour glycolysis initiates a cascade that progressively disables antitumour T-cell immunity: first, competition for glucose deprives effector CD8^+^ T cells of metabolic fuel; second, the resulting lactate accumulation acidifies the microenvironment and directly suppresses T-cell activation and cytolysis; third, accompanying hypoxia further reduces T-cell survival and dampens production of pro-inflammatory cytokines, consolidating an immunosuppressive niche. In parallel, the epitranscriptomic mark N^6^-methyladenosine (m^6^A) orchestrates post-transcriptional control of these immune dynamics: m^6^A readers such as YTHDF1 enhance mRNA translation and stability, whereas YTHDF2 promotes transcript decay. Functionally, YTHDF1 in dendritic cells favours the translation of immunosuppressive programs that curtail cross-presentation, thereby limiting the priming of tumour-specific CD8^+^ T cells (85). Conversely, YTHDF1 deficiency in dendritic cells restores this axis by improving cross-priming and boosting type I interferon production via STING activation, which together counteract glycolysis-driven T-cell dysfunction. Thus, metabolic reprogramming and m^6^A-dependent regulation act in a linked, stepwise manner to shape immune exclusion and modulate responsiveness to immune checkpoint blockade (86, 87). Tumour-intrinsic YTHDF1 also promotes immune evasion by driving lysosomal degradation of MHC-I and antigens. In immunocompetent mice, YTHDF1 loss in tumour cells suppresses tumour growth by enhancing infiltration of CD8^+^ and CD4^+^ T cells, restoring immune surveillance. This occurs via reduced lysosomal proteolysis, improved antigen presentation, and activation of immune pathways (88). While these findings reveal YTHDF1 as a key modulator of tumour immune evasion, further research is needed to elucidate how tumour–TME interactions regulate its expression (89).

### 2.6 Tumour–immune microenvironment and chemokine signalling

#### 2.6.1 Loss of 9q34.3 sensitizes tumours to ICIs response

The most substantially changed cytoband was 9q34, which was deleted at a frequency of 44.4% in responders compared to 30.5% in non-responders. Loss of 9q34 was therefore related to sensitivity to ICIs (90). By decreasing the tumour necrosis factor (TNF) cytotoxicity threshold and enhancing T cell-mediated tumour cell death, *TRAF2* deletion improves ICIs efficacy in the overall pan-cancer cohort. In reality, the majority of cases had complete deletions of chromosome 9. Numerous tumour suppressor genes are located on chromosome 9, but *CDKN2A* (9p21.3) deletion in particular is under significant positive selection and is correlated with aggressive tumour growth in a variety of tumour types. These findings support an evolutionary theory in which loss of the entire chromosome 9 occurs as a driving event early in tumour progression, but subsequently results in collateral immunotherapy sensitivity presumably as a result of loss of 9q34 (91).

#### 2.6.2 Single-cell RNA-seq identifies *CXCL13* and *CCR5*

On *ex vivo* CD8^+^ TILs from a treatment-naive NSCLC patient, sorted according to positivity for a clonal neoantigen (MTFR2) multimer, we performed single-cell RNA sequencing (RNA-seq). The second highest differentially expressed gene was CXCL13 and showed the most pronounced selective expressions in ICIs responders. In addition, it emphasizes that CXCL13 expression may be a characteristic of clonal neoantigen-reactive CD8^+^ TILs that correlates with ICIs outcome in a pan-cancer cohort. CCR5, a chemokine receptor essential for T cell motion inside draining lymph nodes and tumour tissues, was the gene with the next-highest level of expression among responders (92). Together, these findings suggest that CXCL13 and CCR5 expression may serve as hallmarks of clonally expanded, neoantigen-specific CD8^+^ T cells, reflecting an active and spatially organized antitumour immune response associated with favourable ICI outcomes.

## 3 Extrinsic mechanism of resistance to immune checkpoint inhibitors

**Figure 2:**
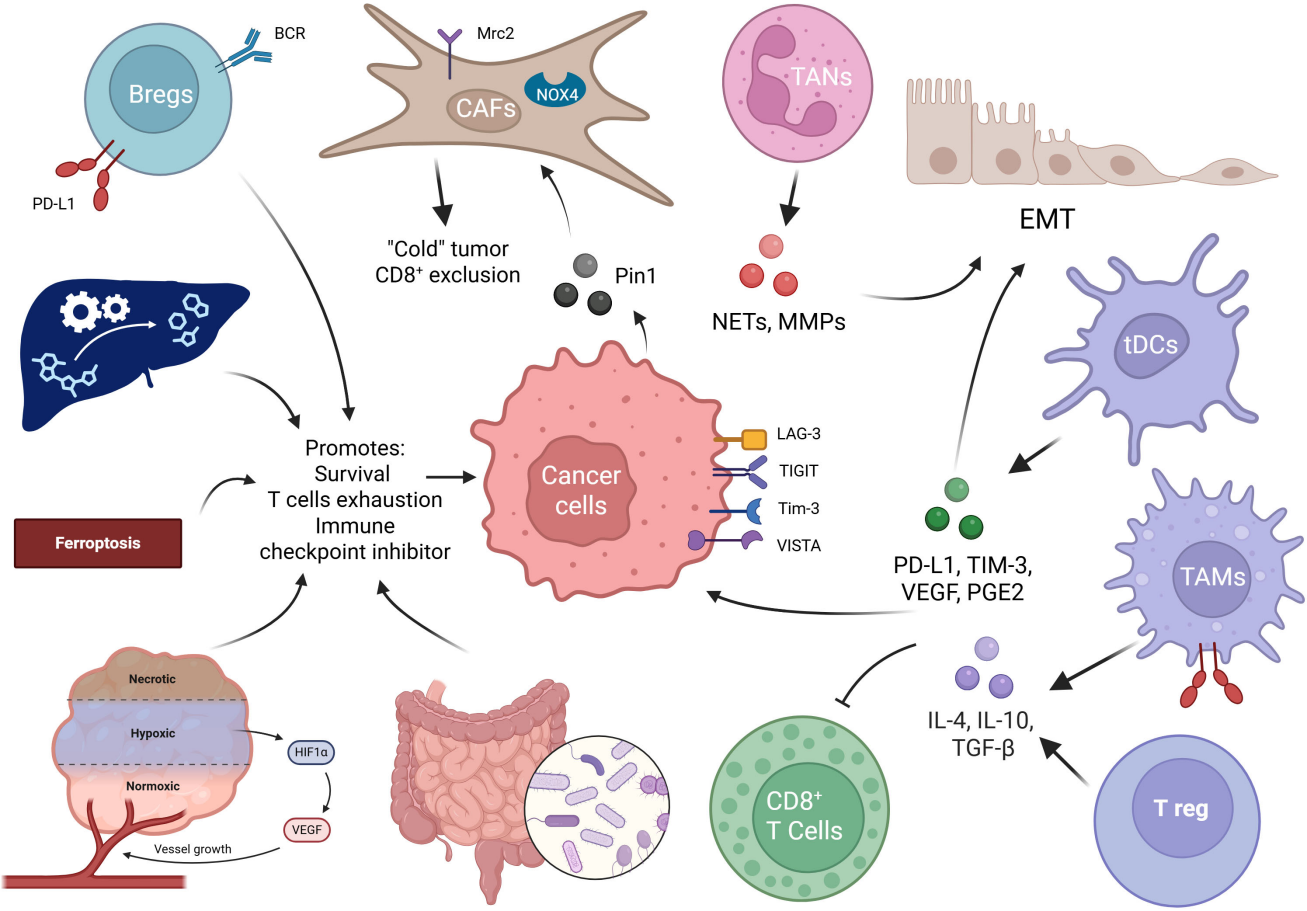
Schematic overview of extrinsic mechanisms in the tumour microenvironment (TME) that promote immune suppression and support cancer-cell persistence. In the center, they have cancer cell expresses immune checkpoints and ligands that inhibit effector lymphocytes. Surrounding components and their principal actions are indicated by arrows. Regulatory B cells (Bregs) secrete immunomodulatory mediators (e.g., IL-10) and antibodies that dampen anti-tumour responses. Cancer-associated fibroblasts (CAFs), including NOX4-expressing subsets, remodel extracellular matrix and release soluble factors/extracellular vesicles that promote tumour survival and immune evasion. Tumour-associated neutrophils (TANs) secrete pro-tumoural mediators that disrupt epithelial integrity and recruit suppressive myeloid cells. Tolerogenic dendritic cells (tDCs) and tumour-associated macrophages (TAMs) present antigen inefficiently and produce immunosuppressive cytokines and ligands, impairing cytotoxic responses and fostering regulatory cell expansion. Regulatory T cells (Tregs) suppress CD8^+^ T-cell activity via contact-dependent mechanisms and inhibitory cytokines (notably IL-4, IL-10, TGF-β). CD8^+^ cytotoxic T lymphocytes are the primary anti-tumour effectors but are functionally inhibited by combined checkpoint engagement and soluble/ cellular suppression. Additional extrinsic influences shown include modulation by the gut microbiota, hypoxia-driven HIF-1α, and the role of ferroptosis in altering antigen presentation and immune activation. Arrows indicate predominant directions of recruitment, activation, polarization and T-bars indicated suppression.

**Figure 2** and **Table 2** summarized the major extrinsic mechanism of resistance.

**Table 2:** Summary of the main extrinsic resistance mechanisms to immune checkpoint inhibitors.

Mechanism	Main pathway	Primary resistance	Acquired resistance	Predictive value	Target	Number of clinical trial	Phase	Type of cancer
MDSCs	Immuno-suppression	+	+	X				
TAMs	Immuno-suppression	++	+	0				
TANs	Immuno-suppression	+	-/+	0				
Tregs	Immuno-suppression	++	+	X	CD25	13	3	Haematological malignancies, neuroblastoma, melanoma, lung cancer, HNC
DCs	Immune modulation	++	+	X	STING / TLR Vaccine Oncolytic virus FLT3L CD40	TLR: 1 Vaccine: 683 Oncolytic virus: 65 FLT3L: 5 CD40: 24	1 3 3 2 2	Pan cancer
CAFs	Immune modulation	+	-/+	0	TGF-ß	TGF-ß: 17	3	Pan cancer
Alternative Immune checkpoints	Immune modulation	+	++	X	LAG-3 TIGIT Tim-3 BTLA VISTA	LAG-3: 39 TIGIT: 18 Tim-3: 10 BTLA: 1 VISTA: 0	3 3 2 1	Pan cancer
Phenotype plasticity	Immune modulation	-	+	X	TGF-ß IL-6 IL-8 IL-17 IL-35 GM-CSF	TGF-ß: 17 IL-6: 8 IL-8: 6 IL-17: 0 IL-35: 0 GM-CSF: 183	3 2 2 3	Pan cancer
Ferroptosis	Metabolism	+	-/+	0	FSP1 DHODH			
Hypoxia	Metabolism	+	+	0	VEGF	VEGF: 112	4	Pan cancer
Microbiota	Metabolism	+	-	X	FMT Specific capsules	35	4	Pan cancer
Glucose metabolism	Metabolism	+	-/+	0	MCT1 → AZD3965 OXPHOS → Metformin	AZD3965: 0 Metformin: 9	2	Pan cancer
Others metabolism	Metabolism	+	-/+	0	Tryptophan Glutamine	Tryptophan: 1 Glutamine: 3	2 2	NSCLC, HCC, CRC

MDSCs, Myeloid-Derived Suppressor Cells; TAMs, Tumour-Associated Macrophages; TANs, Tumour-Associated Neutrophils; Tregs, Regulatory T cells; DCs, Dendritic Cells; CAFs, Cancer-Associated Fibroblasts; TGF-β, Transforming Growth Factor beta; IL, Interleukin; GM-CSF, Granulocyte-Macrophage Colony-Stimulating Factor; LAG-3, Lymphocyte Activation Gene 3; TIGIT, T cell Immunoreceptor with Ig and ITIM domains; TIM-3, T cell Immunoglobulin and Mucin domain 3; BTLA, B and T Lymphocyte Attenuator; VISTA, V-domain Ig Suppressor of T cell Activation; STING, Stimulator of Interferon Genes; TLR, Toll-Like Receptor; FSP1, Ferroptosis Suppressor Protein 1 (AIFM2); DHODH, Dihydroorotate Dehydrogenase; VEGF, Vascular Endothelial Growth Factor; FMT, Fecal Microbiota Transplantation; MCT1, Monocarboxylate Transporter 1; OXPHOS, Oxidative Phosphorylation; NSCLC, Non-Small Cell Lung Cancer; HCC, Hepatocellular Carcinoma; CRC, Colorectal Cancer.

### 3.1 Immunosuppression

#### 3.1.1 Myeloid-derived suppressive cells

MDSCs altered immune cells, leading to immunosuppression. There are two main subsets of MDSCs: monocytic MDSCs (M-MDSCs), derived from monocyte precursors, and polymorphonuclear or granulocytic MDSCs (PMN-MDSCs), originating from immature neutrophils. Both arise from aberrant myelopoiesis under chronic inflammation or tumour-driven signals and exert potent immunosuppressive effects within the tumour microenvironment (90, 93). Additionally, the expression of immunosuppressive checkpoints enhanced the epithelial-mesenchymal transition (EMT) transcriptional factors, including as Snail and Twist1. The establishment of the pre-metastatic niche, which may endure in distant organs for up to 2 weeks after main operations, may depend on MDSCs. When compared to presurgical MDSCs, these persisting postsurgical MDSCs in the NSCLC displayed a more potent immunosuppressive activity (94, 95). In the clinic, an increase in MDSCs was associated with tumour progression, decreased ICIs efficacy, and worse outcomes. For instance, patients with hepatocellular carcinoma had considerably higher levels of MDSC in blood compared to healthy controls (96). In patients with refractory diffuse large B-cell lymphoma (DLBCL), circulating levels of MDSCs may serve as robust prognostic biomarkers for OS at two-year follow-up (97). Additionally, in the mouse model, tumours responding to ICIs have increased CD8^+^ T cells and lower Gr-1^+^CD11b^+^ MDSCs during the early stage of the therapy. Beyond MDSCs, both circulating and tumour-infiltrating myeloid populations—including tumour-associated macrophages (TAMs) can serve as dynamic predictive biomarkers, at baseline and throughout ICI therapy, across multiple cancer types (98).

#### 3.1.2 Tumour-associated macrophages

TAMs constitute the majority of immune infiltrating cells in the TME and control the immunosuppressive TME (99). They usually migrate into tumour tissues as monocytes. Additionally, tissue-resident macrophages migrate toward hypoxic and necrotic regions within tumours, where they are exposed to tumour-derived signals that promote their polarization into TAMs. Most cancer patients, including those with breast cancer, hepatocellular carcinoma, NSCLC and other malignancies, have a bad prognosis in proportion to the level of tumour-infiltrating TAMs (100, 101).

The two primary subtypes of macrophages are immunosuppressive M2-macrophages (alternatively activated) and inflammatory M1-macrophages (classically activated) (102). M1 macrophages exert anti-tumour functions by producing cytotoxic mediators, recruiting effector leukocytes, and mediating the clearance of tumour cells via phagocytosis, thereby contributing to tumour elimination. But it has been shown that M2-macrophages express co-inhibitory molecules such PD-L1 and release anti-inflammatory cytokines like IL-10 (103). Dual inhibition of anti PD-1 in cholangiocarcinoma, by alleviating immunosuppressive constrains within the tumour microenvironment (104).

TGF-β has been found to have an negative impact on anti-PD-1/PD-L1 by preventing T-cell activation and PD-L1 expression (105). Through phosphorylation of the Smad2/3 protein and suppression of mitochondrial respiration, TGF-β derived from TAMs reduces the expression levels of IFN-γ and Granzyme B in T cells, inhibiting T-cell activity (106, 107). In addition to encouraging T-cell exclusion and the establishment of regulatory T cells (Tregs) (108), elevated TGF-β in the TME also prevented the development of the Th1 effector phenotype in colorectal cancer (109). By augmenting the expression of PD-L1, PGE2 can suppress T-cell activation and function (110, 111). Both MDSCs and TAMs can produce PGE2. The level of PGE2 in the TME is regulated by the expression of COX-2 and microsomal PGE2 synthase 1 (mPGES1). Through the COX-2/mPGES1/PGE2 pathway, TAMs can promote the expression of PD-L1 in tumour-infiltrating myeloid cells in bladder cancer, which excludes CD8^+^ T cells (112). Similar to this, PGE2 activates the PI3K-AKT-mTOR pathway in ovarian cancer cells to increase PD-L1 expression (113). To further encourage the development of Tregs from naive T cells, PGE2 can promote the expression of Foxp3 (114).

Exosomes have been implicated as mediators of cell–cell communication that promote tumour initiation and invasion within the TME (115). By activating the PI3K-AKT-mTOR pathway via apolipoprotein E, exosomes can enhance cancer cell migration. Exosomal microRNAs have been shown to regulate gemcitabine resistance, and M2 macrophage-derived exosomes specifically contribute to this drug resistance (116). Likewise, miRNAs carried by M2-macrophages-derived exosomes influence responses to anti-PD-1/PD-L1. In glioma, miR-21 is overexpressed and correlates with reduced CD8^+^ T-cell infiltration; inhibiting exosomal miR-21 not only lowers TGF-β1 levels—thereby preventing immune evasion by glioma cells—but also enhances CD8^+^ T-cell proliferation and cytotoxic activity. Another pan-cancer study demonstrated that combining miR-21 deletion with anti-PD-1 yields superior antitumour efficacy compared to either approach alone (117). Furthermore, *in vivo*, exosomal miR-155-5p from M2-macrophages can induce IL-6 production by tumour cells, thereby dampening the T-cell immune response (118).

#### 3.1.3 Tumour-associated neutrophils

TANs exhibit high plasticity within the TME, shifting from pro-inflammatory (anti-tumour) to immunosuppressive (pro-tumour) phenotypes as tumours progress (90, 119, 120). In a TGF-β–rich TME, TANs adopt an N2-like state that suppresses T cell activity through the secretion of arginase-1 (Arg-1), ROS, and NO. They also express PD-L1 and immunosuppressive cytokines, further dampening anti-tumour immunity (121, 122). In addition to immune suppression, TANs contribute to resistance by remodelling the TME. Releasing neutrophil extracellular traps (NETs) that promote metastatic seeding, matrix metalloproteinases (MMPs) that facilitate extracellular matrix degradation and invasion, and pro-inflammatory mediators that sustain chronic inflammation, angiogenesis, and immune evasion. Moreover, PGE2 from TANs amplifies inflammatory gene expression, while pro-angiogenic factors like VEGFA and S100A8/9 support immune exclusion (123). Importantly, TGF-β blockade can reprogram TANs toward an anti-tumour phenotype, offering a potential strategy to overcome immune resistance.

#### 3.1.4 Regulatory T cells

Regulatory T cells (Tregs) are a specialized subset of CD4^+^ T cells characterized by CD4^+^CD25^+^Foxp3^+^CD127^low/–^ expression and are broadly classified into two groups: thymus‐derived (natural) Tregs (nTregs or tTregs) and peripherally induced Tregs (iTregs or pTregs). In the healthy thymus, nTregs develop via cell–cell interactions and establish self‐tolerance— a process regulated in part by NF-κB. In contrast, iTregs arise from naïve peripheral CD4^+^ T cells upon stimulation by tumour antigens, TME‐derived factors such as TGF-β, and other soluble mediators. Once generated, iTregs suppress antitumour immunity by inhibiting effector T cells, NK cells, and dendritic cells, thus facilitating tumour progression.

While Tregs maintain immune homeostasis, they also dampen antitumour responses, acting as a double‐edged sword. Treg‐derived IFN-γ can paradoxically enhance ICIs efficacy, yet intra‐tumoural Tregs correlate with poor prognosis (124, 125). Tregs secrete anti‐inflammatory cytokines (TGF-β and IL-10) that blunt immune activation and express membrane‐bound TGF-β to directly inhibit CD8^+^ T cell and DC function and they employ granzyme–perforin pathways to lyse effector lymphocytes (124, 125). PD-1^+^ effector regulatory T (eTreg) cells are highly proliferative, as indicated by Ki-67 expression, and exhibit potent immunosuppressive activity *in vitro*. Notably, PD-1 blockade further enhances their suppressive capacity under *in vitro* conditions (126). Through VEGF/VEGFR signalling, Tregs also promote angiogenesis. Moreover, Treg‐mediated consumption of IL-2 deprives effector CD8^+^ T cells of this critical growth factor, contributing to T cell exhaustion. Therapeutically, the use of permissive anti-CD25 antibodies selectively deplete Tregs while preserving effector T-cell (Teff) function. These antibodies exploit the high and sustained expression of CD25 on Tregs to induce antibody-dependent cellular cytotoxicity (ADCC), thereby reducing immunosuppressive activity within the tumour microenvironment. Importantly, they are designed not to block IL-2 binding, thus maintaining IL-2–mediated stimulation of effector T cells, which express CD25 only transiently and at lower levels, rendering them less susceptible to ADCC. By disrupting metabolic pathways, Tregs further undermine effector cell fitness and antitumour activity (127). In NSCLC, Ki67^+^ Tregs showed positive correlation with progression before and during the treatment (128).

### 3.2 Immune-modulation

#### 3.2.1 Tumour-associated dendritic cells

Tumour-associated DCs (tDCs) are reprogrammed by tumour-derived signals into immunosuppressive subsets collectively referred to as regulatory DCs. These include plasmacytoid (pDCs), conventional (cDCs), and monocyte-derived DCs (moDCs) whose normal immunostimulatory function are subverted to support tumour immune evasion (129). This functional shift impairs cytotoxic T cell activation and fosters regulatory T cell expansion. Through the secretion of immunosuppressive mediators—PD-L1, TIM-3, VEGF, IL-10, TGF-β, and PGE2—tDCs inhibit CD8^+^ T cell responses (130). They also promote tumour progression via angiogenesis and genomic instability. Subtype-specific functions contribute further: pDCs stimulate IL-10 production from CD4^+^Foxp3^−^ T cells, cDCs enhance Th2, Th17, and Treg differentiation, while moDCs secrete TNF, IL-6, and IL-12, thereby sustaining tumour progression via chronic inflammation (131). CCL19^+^ DC could be helpful to predictive response to anti PD-L1 in TNBC (132).

#### 3.2.2 Cancer-associated fibroblasts

Cancer-associated fibroblasts (CAFs) comprise a major component of the breast TME and, beyond their established roles in promoting tumour growth and metastasis, exert potent immunosuppressive effects. CAFs foster an immunologically “cold” TME, and targeting CAFs or their signalling pathways can enhance sensitivity to ICIs (133, 134). For example, inhibition of the ROS-generating enzyme NADPH oxydase 4 —which is upregulated in CAFs—promotes CD8^+^ T-cell infiltration and improves ICIs responsiveness (135). In pancreatic ductal adenocarcinoma, Pin1 acts as a central regulator of stromal remodelling by driving TGF-β–dependent activation of CAFs into myofibroblasts. This process sustains a dense desmoplastic matrix that reinforces immune exclusion and therapeutic resistance. Pharmacological inhibition of Pin1 reprograms the fibroblastic compartment toward a less tumour-supportive phenotype, attenuates desmoplasia, and restores sensitivity to PD-1 blockade (136).

Jenkins et al. used paired syngeneic mouse mammary tumour models to show that high CAF density correlates with resistance to combined CTLA-4 and PD-L1 blockade. Transcriptomic, flow-cytometric, and histopathologic analyses revealed that CAF-rich tumours exhibit a CD8^+^ T-cell–excluded, immunologically cold phenotype. Genetic deletion of the CAF receptor Endo180 (Mrc2)—which is predominantly expressed on myofibroblastic CAFs—reduced α-Smooth Muscle Actin (SMA)^+^ CAFs and inhibited tumour growth *in vivo*. In co-implantation studies, only wild-type CAFs (not Endo180-deficient CAFs) impaired intra-tumoural CD8^+^ T-cell infiltration. Endo knockout mice displayed increased CD8^+^ T-cell infiltration and enhanced ICIs efficacy. Clinically, high Endo180 mRNA levels predicted poor responses to anti PD-1 in melanoma patients, underscoring the therapeutic potential of targeting specific CAF subpopulations in breast and other CAF-rich cancers (137).

Despite these advances, CAF research faces three major challenges: (1) syngeneic mouse models fail to capture the genetic and microenvironmental heterogeneity of human tumours; (2) co-implantation of *in vitro*–cultured fibroblasts with tumour cells does not fully recapitulate the phenotype of *in vivo* CAFs; and (3) CAFs themselves are a heterogeneous population with distinct subtypes that can both promote and restrain tumour growth, including subsets with tumour-suppressive functions (138).

#### 3.2.3 Other immunosuppressive cells in the TME

Beyond tDCs and Tregs, additional immunosuppressive cells contribute to immune evasion. Regulatory B cells or Bregs dampen anti-tumour responses via IL-10 secretion and PD-L1 expression (139, 140), opposing the potential tumour-suppressive role of conventional B cells. Tumour-associated mast cells are also implicated in disease progression, particularly in later stages. By producing MMP9, growth factors, and proteolytic enzymes, they facilitate angiogenesis, invasion, and metastasis (141). High mast cell infiltration in tumours is frequently associated with poor prognosis, underscoring their clinical relevance in ICIs resistance.

#### 3.2.4 Immunosuppressive cytokines in the tumour microenvironment

Cytokines such as TGF-β, VEGF, IL-4, and IL-10 contribute significantly to resistance to ICIs by impairing CD8^+^ T cell activity and promoting immunosuppressive cell recruitment (142). TGF-β, produced by various TME components including cancer cells, fibroblasts, TAMs, and Tregs, suppresses cytotoxic T cell function by downregulating perforin, granzymes, and IFN-γ via the SMAD pathway, while promoting EMT and immune evasion in CRC through the USF2/S100A8 axis (143). It also facilitates the accumulation of suppressive populations like TAMs and Tregs. Similarly, VEGF—upregulated in the hypoxic CRC microenvironment—promotes angiogenesis and dampens immune responses. In microsatellite-stable colorectal cancer, VEGF-A extends beyond its angiogenic role by inducing TOX expression in tumour-infiltrating T cells. This transcriptional reprogramming enforces a stable exhausted phenotype, contributing to immune evasion and resistance to PD-1 blockade (144). Anti-VEGF therapies have been shown to remodel hypoxia and restore CD8^+^ T cell functionality, enhancing IFN-γ and TNF-α production (145).

Several additional cytokines sustain tumour-induced immunosuppression. IL-6, abundantly produced by tumour cells, fibroblasts, and myeloid populations, activates the JAK/STAT3 pathway, promoting MDSCs differentiation, inhibiting DC maturation, and upregulating PD-L1 on both tumour and immune cells. Elevated IL-6 levels correlate with resistance to ICIs and reduced CD8^+^ T-cell infiltration in several cancers (146). Combined IL-6/PD-L1 blockade restores T-cell activity and enhances antitumour immunity in preclinical models (147).

IL-8, secreted by tumour and stromal cells, recruits neutrophils and PMN-MDSCs via CXCR1/2, driving immune exclusion, angiogenesis, and suppression of effector T-cell trafficking. High IL-8 expression predicts poor response to PD-1 blockade and reduced OS (148).

IL-17, mainly produced by Th17 and γδ T cells, promotes chronic inflammation and tumour growth through NF-κB and STAT3 activation, stimulating fibroblast proliferation, angiogenesis, and myeloid recruitment (149).

IL-35, a heterodimeric cytokine secreted by Tregs and regulatory B cells, exerts potent immunosuppressive effects by inhibiting effector T-cell proliferation and IFN-γ production, while expanding Tregs and MDSCs—thereby establishing a self-sustaining suppressive circuit (150).

Finally, GM-CSF, although critical for antigen-presenting cell activation, can be hijacked by tumour cells to drive the differentiation of MDSCs or M2 under the influence of IL-6 and PGE_2_ (151)tIL-23 and IL-1β further contribute to the establishment of a chronic, pro-inflammatory yet immunosuppressive state that promote tumour progression and impairs ICIs efficacy.

These cytokines orchestrate a complex, interconnected signalling network that remodels the TME, inhibits CD8^+^ T-cell function, and sustains the expansion of suppressive immune subsets. Therapeutic strategies targeting cytokine axes—such as TGF-β inhibitors, anti–IL-6R antibodies, or CXCR1/2 antagonists—represent promising approaches to reprogram the TME and improve responses to ICIs (152, 153).

### 3.3 Immune checkpoints and phenotypic plasticity in immune resistance

#### 3.3.1 Upregulation of alternative immune checkpoints

Other immune checkpoints—such as TIM-3, LAG-3, and VISTA—become upregulated and represent a key tumour-extrinsic pathway driving AR. During tumour progression, checkpoint expression increases sequentially: PD-1 rises early, whereas LAG-3 and BTLA appear later (154) (155). BTLA contains both inhibitory motifs (ITIM and ITSM) and the activating Grb2-binding motif (156, 157). Ritthipichai et al. found that CD8^+^BTLA^+^ tumour-infiltrating lymphocytes exhibit stronger antitumour activity than their BTLA^−^ counterparts, underscoring the need to clarify BTLA’s role in AR. Moreover, T cells with high PD-1 expression are more profoundly exhausted and less responsive to PD-1 blockade than those with intermediate PD-1 levels. Riaz et al. reported that nivolumab therapy in melanoma enhances the expression of multiple immune inhibitory receptors, reflecting acquired resistance mechanisms. This immunologic pressure drives the expansion of resistant tumour clones, sometimes associated with defects in antigen presentation pathways, such as ß2M (158).

In mouse models of PD-1 inhibitor resistance, Koyama et al. showed that TIM-3 overexpression coincides with resistance despite continued PD-1 inhibitor binding to T cells; blocking TIM-3 restored sensitivity but triggered subsequent CTLA-4 and LAG-3 upregulation (159). Similary, Shayan et al. identified elevated TIM-3 expression in two clinical cases of acquired resistance to PD-1 blockade in patients with head and neck cancer (160). Mechanistically, they further demonstrated that PD-1 inhibition promotes TIM-3 upregulation through activation of the PI3K–Akt–mTOR signalling pathway Recently, the TIM-3/Galectin-9 axis has been implicated in both primary resistance and AR to PD-1 blockade in lung cancer patients (161).

Huang et al. found in preclinical studies that inhibition of any one checkpoint among PD-1, LAG-3, or CTLA-4 induces compensatory upregulation of the other two, thereby limiting the efficacy of ICIs in monotherapy in metastatic ovarian cancer (162). In a cohort of NSCLC patients with AR to ICIs, matched pre- and post-treatment samples from eight individuals revealed TIM-3 overexpression in three cases—and all three also co-upregulated LAG-3—while five showed increased LAG-3 alone. LAG-3 binds MHC-II, and in MHC-II–positive tumours, the inhibitory receptor FCRL6 is similarly elevated in resistant specimens (163). Importantly, overexpression of TIM-3 and LAG-3 was linked specifically to AR rather than primary resistance. Gao et al. first reported VISTA upregulation in prostate cancer patients treated with CTLA-4 inhibitors (164). In a separate melanoma cohort of twelve patients with AR to ICIs, eight exhibited VISTA overexpression.

First identified by Yu et al., TIGIT acts as a rheostat inhibiting T-cell activation and is expressed primarily on NK cells, Tregs, and memory T cells (165). Preclinical data indicate that TIGIT blockade can prevent NK-cell exhaustion and elicit robust antitumour responses (166). In an early phase II trial, combining TIGIT inhibition with anti-PD-L1 therapy improved objective response rate and median progression-free survival compared to anti PD-L1 alone in the first-line setting (167). In NSCLC, PD-1 and TIGIT cooperate to dampen CD226 (DNAM-1)–mediated co-stimulatory signalling through distinct mechanisms. PD-1 inhibits CD226 activity by recruiting the phosphatase SHP2 to its intracellular domain, leading to dephosphorylation and functional inactivation of CD226. In contrast, TIGIT acts extracellularly by competing with CD226 for shared ligands such as PVR (CD155), thereby preventing CD226 engagement and downstream activation of effector T cells. The importance of CD226 in regulating anti-tumour responses is demonstrated in mouse tumour models where use of a CD226-neutralizing monoclonal antibody (mAb) abrogates the efficacy of combining mAbs against PD-L1 and TIGIT (168).

#### 3.3.2 Phenotypic plasticity

Phenotypic shifts such as trans-differentiation and epithelial-to-mesenchymal transition (EMT) have emerged as key contributors to therapeutic escape. NSCLC-to-small cell lung cancer transformation, classically observed under EGFR-TKI pressure, has also been reported following ICIs (169, 170). This switch is associated with RB1 inactivation and often confers sensitivity to chemotherapy. EMT is characterized by loss of epithelial markers and acquisition of mesenchymal traits. EMT-related transcription factors like Snai1 and SOX2 promote resistance to CD8^+^ T cell-mediated killing and ICIs (171, 172). EMT-persistent tumour cells exhibit stem-like features and hybrid phenotypes, while quasi-mesenchymal cells can shield epithelial populations from immune clearance and resist anti-CTLA-4 (173).

### 3.4 Metabolism

#### 3.4.1 Ferroptosis and immunosuppression

Ferroptosis is an iron-dependent form of cell death initiated by the Fenton reaction (H_2_O_2_ + Fe²^+^ → OH), leading to lipid peroxidation, irreversible membrane damage, and ultimately cell death, which is often associated with an immunosuppressive tumour microenvironment. Tumour cells undergoing programmed cell death (PCD) release a variety of inflammatory mediators, chemo-attractants, and intracellular components that remodel the local immune microenvironment. In an analysis of 1,750 gliomas across four cohorts, ferroptosis emerged as the most enriched form of PCD and correlated strongly with tumour progression, poor prognosis, and immunosuppression.

Ferroptosis is an iron-dependent, oxidative cell death characterized by lethal accumulation of reactive oxygen species and lipid peroxides. To clarify how ferroptosis influences antitumour immunity, a stepwise evaluation of immune activity was performed. In glioblastoma (GBM) patients, high ferroptosis scores were associated with increased antigen presentation, immune cell trafficking, and overall immune infiltration, along with elevated expression of immune checkpoint molecules compared to low-score tumours. Yet paradoxically, effector immune functions remained suppressed. This apparent contradiction is consistent with the “immunity tidal model,” in which the simultaneous upregulation of both co-stimulatory and co-inhibitory immune checkpoints fosters an immunosuppressive tumour phenotype (174). Using the xCell algorithm, only myeloid populations were enriched in high-ferroptosis GBM, whereas lymphoid cells were reduced. In particular, infiltration by immunosuppressive subsets —Tregs, neutrophils, and M2-macrophages— was associated with elevated ferroptosis, implicating macrophages as key mediators of ferroptosis-driven immunosuppression. Primary glioma spheres (GSC22 and GSC40) treated with the ferroptosis inhibitor ferrostatin-1 for 48 hours, and their conditioned medium was subsequently applied to macrophage migration assays. Ferrostatin-1-treated spheres markedly reduced THP-1-derived macrophage migration, despite, ferrostatin-1 alone having no direct effect on macrophage motility—suggesting that the inhibitor modulates the paracrine signalling profile of treated glioma spheres. Moreover, macrophages exposed to conditioned medium upregulated M1-macrophages markers (CD11c, CD80) and downregulated M2-macrophages markers (CD163, LYVE1). Comparable results were seen in PBMC-derived macrophages. Conversely, conditioned medium from glioma spheres treated with the ferroptosis inducer erastin enhanced macrophage chemotaxis and skewed polarization toward an M2-like phenotype (175).

#### 3.4.2 Hypoxia

Tumour hypoxia has long been recognized as a key driver of immune evasion, but the molecular mechanisms underpinning this effect have remained insufficiently defined. In a comprehensive study, Ma et al. employ an *in vitro* model of triple-negative breast cancer to show that hypoxia suppresses the expression of immune effector genes in both CD8^+^ T cells and NK cells, contributing to functional exhaustion and resistance to ICIs. Mechanistically, they identify a HIF1α–HDAC1 axis, acting in cooperation with pre-bound PRC2, that mediates chromatin remodelling at effector loci. This epigenetic reprogramming reduces H3K27 acetylation while increasing H3K27 trimethylation, leading to transcriptional silencing of cytotoxic programs and a dysfunctional immune phenotype. These observations are reinforced by *in vivo* experiments using syngeneic and humanized mouse models, where blockade of either HIF1α or HDAC1 restores effector gene expression and enhances responsiveness to anti PD-1. Hypoxia also dampens type I interferon responses and expression of PD-L1/PD-L2 in tumour cells, further contributing to immune evasion. Notably, restoring HIF1α-regulated chromatin accessibility reinstates IFN-γ production in CD8^+^ T and NK cells and promotes tumour regression in combination with anti PD-1. While hypoxia’s roles in tumour growth and therapy resistance are well documented, its contribution to immune suppression is increasingly evident (176).

Conflicting results in prior studies have fuelled debate around hypoxia’s immunological consequences. Some mouse studies suggest that hypoxia enhances T cell cytotoxicity (177, 178), while others show it promotes exhaustion and impairs NK cell infiltration (179, 180). Overexpression of HIF1α in murine T cells reduces their antitumour efficacy, and clinical data indicate that hypoxia-associated transcriptional signatures correlate with T cell dysfunction and reduced response to anti PD-1 in melanoma and HNSCC patients (181, 182).

Ma et al. show that in human immune cells, HIF1α-dependent epigenetic repression of effector genes is a dominant mechanism of dysfunction. In parallel, hypoxia upregulates co-inhibitory receptors such as TIM-3 and TIGIT in a HIF1α-independent manner, suggesting a multifaceted process involving both transcriptional and metabolic pathways.

In another study, Bannoud et al. provide mechanistic insight into how hypoxia, along with VEGF-A, promotes the terminal differentiation of exhausted PD-1^+^TIM-3^+^CXCR5^+^ CD8^+^ T cells at the expense of progenitor-like PD-1^+^TIM-3^−^ subsets. Despite retaining effector molecule expression (e.g., TNF-α, IFN-γ, GZMB), these terminally exhausted cells adopt a proangiogenic transcriptional profile, with VEGF-A being the primary hypoxia-induced factor. This shift underscores the reciprocal relationship between hypoxia-driven vascular remodelling and immune exhaustion (183).

Hypoxia also upregulates TIM-3 expression, a hallmark of terminally exhausted T cells, and skews the CD8^+^ T cell pool toward ICIs-refractory subsets (184, 185). Anti PD-1 may preferentially act on progenitor exhausted T cells (TIM-3^−^, TCF1^+^, PD-1^+^); thus, promoting their maintenance could enhance therapeutic outcomes.

Together, these findings position hypoxia as a central regulator of immune resistance through both epigenetic and angiogenic programs. Targeting HIF1α or VEGF-A–mediated pathways, in combination with ICIs, represents a promising strategy to overcome resistance in highly hypoxic tumours (186).

#### 3.4.3 Microbiota

Vetizou et al. demonstrated that the efficacy of anti CTLA-4 in melanoma is shaped by gut microbiota composition, particularly *Bacteroides* spp. In both mice and humans, anti–CTLA-4 responses were linked to T cell reactivity against *B. fragilis* or *B. thetaiotaomicron*. Antibiotic-treated or germ-free mice failed to respond to therapy, but responsiveness was restored by oral *B. fragilis*, immunization with its polysaccharides, or transfer of *B. fragilis*-specific T cells. Fecal transplant from responsive melanoma patients confirmed the role of Bacteroides in mediating therapeutic efficacy. Mechanistically, anti CTLA-4 promotes Bacteroides accumulation in the inner mucus layer, enhancing IL-12–dependent Th1 responses via mucosal dendritic cells. Gut microbiome profiling of ipilimumab-treated melanoma patients identified three enterotypes, distinguished by Bacteroides and *Prevotella* spp. Building on this stratification, fecal microbiota from cluster C patients—enriched in immunostimulatory species such as *Bacteroides fragilis* and *B. thetaiotaomicron*—successfully restored anti–CTLA-4 efficacy in germ-free mice. In contrast, microbiota from cluster B was associated with therapeutic resistance (187).

Sivan et al. demonstrated that commensal microbiota modulates anti-tumour immunity in melanoma-bearing mice. Tumours grew more aggressively in Taconic (TAC) mice compared to Jackson Laboratory (JAX) mice, correlating with reduced tumour specific CD8^+^ T cell responses. Notably, fecal transfer or cohousing with JAX mice normalized tumour growth in TAC mice, implicating microbiota composition as a key regulator. Amplicon sequencing identified Bifidobacterium as a beneficial genus, enhancing intra-tumoural DC function and CD8^+^ T cells priming. Oral Bifidobacterium administration restored T cell responses and improved tumour control, significantly enhancing the efficacy of anti PD-L1 (188).

It is well established that the gut microbiota and its metabolites play a role in immune responses and tumour development. In melanoma, responders’ patients receiving anti PD-1, exhibited significantly higher gut microbiome diversity and greater abundance of *R.* sp*ecies*. Metagenomic and immune profiling revealed enrichment of anabolic pathways and enhanced antitumour immunity in responders (189). By activating TLR4 signalling, Fusobacterium nucleatum promotes the proliferation of colorectal cancer cell lines such as HCT116 and LoVo through NF-κB–mediated upregulation of oncogenic miR-21 (190). In parallel, it facilitates TAM polarization toward the M2 phenotype via the TLR4/NF-κB/S100A9 axis and the miR-1322/CCL20 pathway, thereby enhancing colorectal cancer progression and metastatic potential (191, 192). By interacting with TIGIT, the Fap2 (Fusobacterium adhesion protein 2) protein produced by *F. nucleatum* prevents T cell activation (193). Probiotics, a commensal bacterial family found in the gut are crucial for tumour regression and enhancing the outcomes of immunotherapies. By limiting HDAC activity, *C. butyricum* inhibits CRC proliferation by releasing butyrate into the TME (194). *L. rhamnosus* and *L. casei* can help CD8^+^ TIL infiltrate tumours and release cytotoxic cytokines, which enhances the anti-tumour activity of anti PD-1 drugs (195, 196).

Gut microbiome composition has emerged as a critical determinant of response to ICIs. In a pivotal study, Routy et al. demonstrated that primary resistance to PD-1 blockade in patients with NSCLC and renal cell carcinoma correlated with a marked depletion of *Akkermansia muciniphila* in the gut microbiota. Antibiotic exposure prior to immunotherapy was associated with reduced clinical benefit, underscoring the importance of microbial integrity. Fecal microbiota transplantation (FMT) from responder patients into germ-free or antibiotic-treated mice restored the antitumour efficacy of PD-1 blockade, whereas FMT from non-responders did not. Notably, oral supplementation with *Akkermansia muciniphila* following non-responder FMT reinstated therapeutic efficacy in an IL-12–dependent manner, by promoting the recruitment of CCR9^+^CXCR3^+^CD4^+^ T cells into the tumour microenvironment. These findings highlight *Akkermansia muciniphila* as a key immunomodulatory commensal bacteria capable of enhancing ICI responsiveness through microbiota–immune crosstalk (197).

One pathway that may explain the impact of the microbiome on the response to immune checkpoint inhibitors (ICIs) involves the interaction between microbial cells and myeloid cells. Indeed, the microbiome plays a regulatory role in myelopoiesis and contributes to the homeostasis of neutrophils and monocytes. Certain microbial taxa have been shown to be predictive of treatment response, such as *Bifidobacterium*, whereas others may be implicated in acquired resistance, such as *Bacteroides* spp., particularly following anti–CTLA-4 therapy (198, 199).

#### 3.4.4 Glucose metabolism

CD8^+^ T cells isolated from tumours with high glycolytic activity but limited glucose availability within the TME exhibit a significantly reduced rate of glycolysis. Additionally, they produce significantly less IFN-γ, and these exhausted phenotypes are associated with faster tumour progression. The inhibition of glucose uptake in NK cells had a significant detrimental effect on their effector function. This included lower production of IFN-γ and granzyme B, as well as disrupted adhesion with target cells (200). By stabilizing c-MYC transcription in mouse or PDX models, lncRNAs (long non-coding RNAs) GLCC1 and LINRIS increase tumour glycolysis and growth, corresponding with a poor prognosis in CRC (201, 202). Malignant cells can directly affect T cell glucose metabolism by expressing CD155 and interacting with TIGIT on the T cell surface, which leads to a malfunction in T cell energy use (203, 204).

#### 3.4.5 Other nutrients

Metabolic by-products within CRC microenvironment actively contribute to immune evasion and tumour progression. Lactate promotes angiogenesis via endothelial tube formation and skews macrophages toward an M2 phenotype, while its removal restores CD8^+^ T cell function in acidic TME (205, 206). Tryptophan depletion through IDO1/2 and TDO2-mediated catabolism generates immunosuppressive kynurenine, which induces PD-1 expression in CD8+ T cells via AHR (Aryl hydrocarbon Receptor) activation and promotes Treg polarization (207). Glutamine metabolism further fuels CRC growth, with KRAS-driven SLC1A5 expression enhancing glutaminolysis and activating Wnt/β-catenin signalling to support stemness (208, 209). Glutamine is also essential for T cell proliferation. Lipid accumulation in CRC cells imposes metabolic stress on infiltrating CD8^+^ T cells via CD36-mediated fatty acid uptake, leading to ferroptosis and reduced cytokine production (210). Collectively, these metabolic pathways define an immunosuppressive TME and represent tractable targets for therapeutic intervention.

## 4 Precision approaches for predicting ICI response

### 4.1 Genetic biomarkers

The prognostic relevance of TMB, MSI, and dMMR as predictive biomarkers for ICIs has been well established. High TMB correlates with improved responses to anti CTLA-4 in melanoma and anti PD-1 in NSCLC (211). In patients with dMMR tumours, pembrolizumab demonstrated enhanced response rates and PFS across tumour types. This led to its landmark FDA approval in 2017 for MSI-high or dMMR solid tumours irrespective of tissue origin, based on pooled trial results showing a 39.6% response rate, with 78% achieving durable responses of ≥6 months (212). A second tissue-agnostic approval was granted for high TMB (≥10 mutations/Mb) following KEYNOTE-158, and pembrolizumab became first-line therapy for MSI-high colorectal cancers per KEYNOTE-177. Nonetheless, TMB is not universally predictive. Some patients do not respond despite high TMB, and its association with response may be limited to ICI-naive individuals. Moreover, the need for advanced genomic platforms restricts routine clinical application.

### 4.2 Tumour-infiltrating lymphocytes

Pre-treatment immune profiling has emerged as a critical component in predicting response to ICIs. In metastatic melanoma, a high baseline density of intra-tumoural CD8^+^ T cells is strongly associated with favourable responses to anti PD-1 (213). Additionally, T cell abundance at the invasive tumour margin prior to treatment correlates with improved outcomes. Responders exhibit increased T cell infiltration both at the margin and within the tumour core (214). However, TILs appear less informative for predicting response to anti CTLA-4, likely reflecting its distinct mechanism of action. Notably, early on-treatment TILs levels have shown predictive value for anti CTLA-4 efficacy, as reported by Chen et al., suggesting that serial biopsies during treatment could provide critical insights (214). To improve reproducibility, the International Immuno-Oncology Biomarker Working Group is standardizing TILs assessment protocols (215). Immunoscore is a quantitative immune-based classifier that evaluates the density and spatial distribution of CD3^+^ and CD8^+^ T lymphocytes within the tumour core and invasive margin, providing a robust measure of the host immune response in CRC. Developed by Galon et al., Immunoscore has proven to outperform traditional TNM staging in predicting prognosis, particularly in CRC. Patients with a high Immunoscore (I3–I4) display significantly improved OS compared with those with low scores (I0–I1), reflecting effective cytotoxic infiltration and immune surveillance. In a large international validation study including >2,600 patients, Immunoscore was identified as the strongest prognostic factor for time to recurrence, independent of MSI status or clinicopathological variables (216, 217).

### 4.3 Immune checkpoint molecule expression

Initial studies across cancer types, including melanoma and NSCLC, indicated a significant association between pre-treatment PD-L1 expression and responsiveness to anti PD-1/PD-L1. The Tumour Proportion Score (TPS) and the Combined Positive Score (CPS) are two standardized metrics used to quantify PD-L1 expression and guide eligibility for immune checkpoint inhibitor therapy. TPS represents the percentage of viable tumour cells showing partial or complete membranous PD-L1 staining. In contrast, CPS incorporates both tumour and immune components, calculated as the number of PD-L1–positive tumour cells, lymphocytes, and macrophages divided by the total number of viable tumour cells, multiplied by 100. While TPS is primarily used in non–small cell lung cancer, CPS provides a broader assessment of PD-L1 expression in the tumour microenvironment and is commonly applied in other malignancies such as gastric and head and neck cancers. However, durable responses have also been documented in patients with PD-L1^-^ tumours (218), underscoring the complexity of this biomarker. Variability in PD-L1 assay methodologies and positivity thresholds further complicates interpretation, highlighting the need for assay harmonization.

### 4.4 Soluble immune checkpoints as predictive biomarkers

Soluble immune checkpoints (sICPs), including sPD-1, sPD-L1, sCTLA-4, sLAG-3, sTIM-3, sB7-H3, sBTLA, and sHVEM, have emerged as minimally invasive biomarkers that reflect immune activation and regulatory dynamics in the TME. These soluble forms result from alternative splicing or proteolytic shedding of membrane receptors and ligands, and their circulating levels can fluctuate in response to immunotherapy. Studies have demonstrated that high baseline levels of sPD-L1 are associated with poor response and shorter survival in patients receiving ICI across multiple malignancies, including renal cell carcinoma (RCC). In metastatic RCC cohorts treated with nivolumab, low baseline concentrations of sPD-1, sPD-L1, and sBTN3A1 have been correlated with durable clinical benefit and improved overall survival, while early increases in sPD-L1 levels during therapy have been linked to disease progression (219). Our group work on sBTN2A1 and found that high baseline sBTN2A1 was associated with shorter in a cohort of pretreated advanced RCC (220). Expanding beyond PD-1/PD-L1, soluble forms of LAG-3, TIM-3, and BTLA also show potential prognostic and predictive value, particularly when analysed in combination as part of multiplex immune monitoring. Collectively, sICPs offer a promising avenue for real-time, blood-based assessment of ICI efficacy, complementing tissue-based PD-L1 scoring and transcriptional immune signatures (221–223).

### 4.5 Soluble non-immune biomarkers as predictive biomarkers

Beyond immune-related determinants, non-immune biomarkers are increasingly recognized as key modulators of ICI response. Among them, Kidney Injury Molecule-1 (KIM-1, also known as HAVCR1) has recently gained attention in RCC as a circulating biomarker with both prognostic and potential predictive value. KIM-1, a transmembrane glycoprotein expressed and released by damaged tubular epithelial and tumour cells, is elevated in the serum and urine of RCC patients and reflects tumour burden and tissue injury. Recent studies in metastatic RCC have reported that higher sKIM-1 levels correlate with poorer outcomes under nivolumab, whereas dynamic decreases in responders (224). In the adjuvant setting, elevated baseline sKIM-1 levels were associated with higher recurrence rate, suggesting potential utility as a predictive marker of ICI (225). Ongoing clinical validations indicate that KIM-1 may soon be implemented as a routine biomarker in RCC (NCT01063998).

### 4.6 Microbiome signatures

Microbial ecosystems, both gut and tumour-associated, have emerged as modulators of antitumour immunity and ICIs responsiveness (226, 227). TCGA data further reveal distinct microbial profiles in circulation that differentiate cancer patients from healthy individuals (228). Circulating microbiota signatures may therefore represent a minimally invasive, next-generation class of biomarkers. Lin et al., analysed fecal metagenomes from 1,359 patients with metastatic melanoma, NSCLC, renal cell cancer or hepatocellular carcinoma and they found that gut microbial composition—including bacteria, eukaryotes, viruses, and archaea—differs significantly between ICIs responders and non-responders. Specific trans-kingdom species, such as *F. prausnitzii* and *N. serpens*, were enriched in responders and shown to enhance CD8^+^ T cell activity (229). Circulating CD3^+^HLA-DR^+^ T cells were a prognostic biomarker for ICIs, with higher baseline frequencies linked to less reponses and shorter OS.

Combining CD3^+^HLA-DR^+^ T cell levels with the neutrophil-lymphocyte ratio (NLR) and gut microbiota profiles—particularly *Bacteroides vulgatus* and *Burkholderiales*—, an immune-microbial scores was established and it showed strong predictive value on OS (230).

### 4.7 Emerging biomarkers and strategies

Although TILs and PD-L1 remain informative, they are insufficient alone to capture the complexity of immune resistance. Recent efforts aim to expand the biomarker landscape. For example, expression of checkpoint molecules (PD-1, PD-L1, LAG-3) and T cell markers (CD3, CD4, CD8, FOXP3, GZMB) in early on-treatment biopsies following sequential CTLA-4 and PD-1 blockade has been linked to treatment responsiveness (231). The ongoing AMADEUS trial explores CD8^+^ T cell density as a predictor of benefit from nivolumab monotherapy or combined ICIs. It further aims to stratify tumours into immunologically “hot” versus “cold” categories and identify novel predictive biomarkers but it is also available to separate this in 4 immune infiltration patterns describe the spatial organization of immune cells within the TME and predict immunotherapy response: structured (inflamed) tumours show dense intratumoural infiltration; excluded tumours confine immune cells to the margins; dispersed tumours display irregular immune distribution; and desert tumours lack immune infiltration, reflecting an immunologically “cold” phenotype (232). Exosomes PD-L1^+^, particularly in combination with CD28 expression, has demonstrated high predictive accuracy (AUC = 0.85) (233). Nevertheless, larger cohorts and longitudinal validation are required to confirm its clinical utility in ICIs-treated populations. Several new biomarkers were developed to made a better selection of patients for ICIs, for example in NSCLC received ICIs and chemotherapy, high EGLN3 expression was associated with worth outcome (234). In NSCLC treated with ICIs in monotherapy, Rakaee et al., found that association of Deep Learning Model of IHC and PD-L1 expression could be predictive to better PFS (hazard ratio, 0.56; 95% CI, 0.42-0.76; P <.001) and OS (hazard ratio, 0.53; 95% CI, 0.39-0.73; P <.001) (235).

### 4.8 Obesity and sex

Other, more clinically oriented parameters are also emerging as potential biomarkers of response to immunotherapy. Obesity, defined as a body mass index (BMI) ≥ 30, is a well-established risk factor for cancer and is generally associated with poor outcomes following treatments such as allogeneic hematopoietic stem cell transplantation. However, paradoxically, obesity has been identified as a predictive factor for response to immune checkpoint inhibitors, a phenomenon referred to as the “obesity paradox” (236).

Sex-related differences also contribute to variations in both innate and adaptive immunity. Females tend to exhibit greater activation of macrophages and neutrophils, whereas males show enhanced activation of natural killer (NK) cells. Additional differences include more efficient antigen presentation and a predominance of Th2 immune responses in females. Notably, improved responses to ICIs have been reported in obese male patients (236, 237).

## 5 Discussion

Multiple strategies have emerged to overcome resistance to ICIs, leveraging insights into tumour biology and immune evasion mechanisms.

Tumours with poor immunogenicity often evade ICIs due to impaired antigen presentation. Inhibitors of DNA methyltransferases can restore MHC-I expression and improve antigen visibility to T cells (238). Similarly, combining these agents with histone deacetylase HDAC inhibitors has shown synergistic immune activation in lung cancer preclinical studies (239). Combining ICIs with therapies that correct oncogenic signalling holds promise. For example, adding atezolizumab to BRAF/MEK inhibitors enhances PFS in BRAF-mutated melanoma (240). Inhibitors of PI3K and CDK4/6 have also demonstrated potential to reverse immune evasion in preclinical models (241) (242).

Bispecific antibodies (BsAbs) offer a promising strategy to overcome resistance to ICIs by simultaneously targeting multiple immune pathways or cell populations. In cases of low T cell infiltration or AR, BsAbs can bridge tumour cells to an effector immune cells—for example, by engaging CD3 on T cells and a tumour-associated antigen—thereby bypassing the need for endogenous T cell priming or MHC-I antigen presentation. Furthermore, bispecific formats that concurrently block two inhibitory receptors (e.g., PD-1 and LAG-3 or PD-1 and TIGIT) may more effectively reverse T cell exhaustion than monotherapies or even dual ICIs, especially in tumours exhibiting compensatory upregulation of alternative checkpoints. BsAbs can also be engineered to co-deliver immune costimulatory signals or cytokine payloads directly within the TME, enhancing local immune activation while minimizing immune toxicity. Targeting both PD-1 and LAG-3, Tebotelimab showed a good safety profile with 34% of responses in several recurrent solid or hematological cancer (243). A phase 3 trial showed Cadonilimab, an BsAbs targeting PD-1 and CTLA-4, improved PFS by 4.6 months (HR 0.62 [95% CI 0.49-0.80], p<0.0001) and OS by 4.2 months (HR 0.64 [0.48-0.86], p=0.0011) in first line treatment for patients with cervical cancer (244). Cibisatamab, a BsAbs targeting CEA (Carcinoembryonic Antigen) on tumour cells and CD3, was evaluated in two phase 1 trials (with or without Obinutuzumab and with atezolizumab) for advanced CEA-positive solid tumours. Nevertheless, despite an high grade 3 or 4 adverse events rate (36% or 49%), it showed modest efficacy (ORR 4–7%), with improved response (14%) in MSS-CRC (245).

Blocking angiogenesis can remodel the immunosuppressive TME. Combining anti-VEGF with ICIs has improved outcomes in cancers such as hepatocellular carcinoma (246). Chemo- and radiotherapy can induce immunogenic cell death and enhance T cell responses. In CRC, the combination of pembrolizumab and mFOLFOX6 yielded a median PFS of 8.8 months and an ORR of 56.7% (247). However, benefits may vary with tumour mutations. For instance, *KRAS*-G12D-mutant NSCLC showed improved response to chemo-immunotherapy, likely via CD8+ T cell recruitment through HMGA2-driven chemokine production (248).

Oncolytic viruses (OVs) can enhance tumour immunogenicity by inducing cell lysis and immune activation. Talimogene laherparepvec (T-VEC) combined with ipilimumab improved response rates in melanoma (249). OVs was also tested in CRC in association with durvalumab with or without tremelimumab. Safety profile was acceptable nevertheless median PFS was limited at 2.1 months or 2.3 months in third or more line (250). OVs are also being explored to augment CAR-T cell therapies by enhancing antigen presentation or modifying TME (251).

Cancer vaccines aim to boost antigen-specific T cell responses. Trials with neoantigen vaccines (252) and DC-based therapies, including Flt3L to expand DCs, have shown promise (253). A clinical trial using Flt3L, poly-ICLC, and radiotherapy achieved a 72.7% ORR in low-grade B-cell lymphoma (254). Targeting components of the TME, such as TAMs or vasculature, can enhance ICIs efficacy. Trials with CSF1R inhibitors and VEGFR inhibitors like regorafenib showed modest benefit in MSS CRC (255). Fecal microbiota transplants from ICIs responders to non-responders have improved response rates in melanoma, supporting the microbiome’s role in ICIs resistance (189, 256). Manganese salts, by enhancing DC function and T cell activation, have shown promising early results in ICIs combinations (257). PI3Kβ inhibition is also under evaluation for its ability to boost antitumour immunity while sparing immune cells.

Local treatments (surgery, radiotherapy) can extend ICIs benefit in patients with limited progression. In Keynote-006 study, patients receiving local resection followed by retreatment with pembrolizumab experienced durable responses (258).

Loss of MHC-I or *JAK* mutations disrupt antigen presentation or IFN responses. Strategies such as NK cell therapy, NLRC5 overexpression, and use of BO-112 (a poly I:C analog) have restored immune recognition in preclinical models (259). Pharmacological inhibition of pathways like Wnt/β-catenin, TGF-β, IDO, or adenosine may reverse immune exclusion and improve ICIs response by targeting exclusion and immunosuppression pathways (260). Tumour plasticity, including EMT, can be countered with epigenetic drugs. In melanoma, ferroptosis sensitivity varies with cell state, offering a route to sensitize tumours to ICIs (261). Resistance due to alternate checkpoint upregulation (e.g., LAG-3, TIGIT) can be addressed via combinatorial blockade. TIGIT inhibition has demonstrated synergy with anti-PD-L1 in early trials (166). Combination of fianlimab (an anti-LAG-3) with cemiplimab (an anti PD-1) in advanced melanoma showed promising results for patients treatment naïve or with received previous anti PD-1 in neo-adjuvant or adjuvant setting, the ORR was 63% or 61.5% and median PFS 12.6 months or 12 months, respectively. In patients received previous anti PD-1 in advances setting, ORR 13.3% and median PFS was 1.5 months (262).

Efforts to understand and predict primary or acquired resistance to ICIs may also contribute to improving our understanding of a still poorly characterized phenomenon, hyper progression (HP). HP refers to an acceleration of tumour growth rate following the initiation of ICI therapy. However, its definition remains non-consensual. HP has been reported across all tumour types, with an incidence ranging from 4% to 29%. The underlying mechanisms implicated in HP remain largely hypothetical and include, among others, an enrichment of CD163^+^CD33^+^PD-L1^+^ TAMs in hyper progressive cases, activation of PD-L1^+^ regulatory T cells by ICIs, selection of immune-resistant subclones in patients previously exposed to anticancer therapies, and preferential activation of Th17 cells. In addition, innate lymphoid cells type 3 (ILC3), induced by ICIs, secrete pro-inflammatory interleukins that promote tumour growth. Collectively, these mechanisms overlap with pathways involved in primary resistance to ICIs. Although the incidence of HP may be reduced by combining chemotherapy with ICIs, hyper progression can still occur (263, 264).

Therapeutic resistance to ICI reflects the dynamic co‐evolution of cancer cells and their surrounding milieu. Tumours adapt through alterations in antigenicity and intracellular signalling that blunt T‐cell recognition and effector function. Early clinical and preclinical studies indicate that rational combinations—pairing checkpoint inhibitors with epigenetic drugs, targeted kinase inhibitors, oncolytic viruses, neoantigen vaccines, or microbiome modulators—can recalibrate both tumour‐intrinsic and microenvironmental barriers. Moving forward, leveraging spatially resolved, single‐cell profiling alongside circulating biomarkers will be essential to monitor resistance trajectories in real time and to tailor acquired treatment regimens.

In conclusion, the expanding role of immunotherapy in oncology underscores the need for a deeper understanding of the mechanisms underlying both primary resistance, which leads to early disease progression in a large proportion of patients and varies across tumour types, and acquired resistance, which results in relapse after an initial response. Resistance to immune checkpoint inhibitors (ICIs) may arise from tumour-intrinsic factors, components of the tumour microenvironment, or their dynamic interactions. Elucidating these mechanisms is essential for the identification of novel biomarkers to improve patient selection and treatment monitoring, as well as for the development of new therapeutic strategies. This review highlights the complexity and diversity of resistance mechanisms to ICIs and emphasizes that a comprehensive, such integrative approaches hold promise for durable, personalized immunotherapy.
